# Archaea in Past and Present Geobiochemical Processes and Elemental Cycles

**DOI:** 10.1155/2013/930493

**Published:** 2013-06-16

**Authors:** Michael Hoppert, Martin Krüger, Joachim Reitner, Charles Cockell

**Affiliations:** ^1^Institute of Microbiology and Genetics, Georg-August-University Göttingen, 37077 Göttingen, Germany; ^2^Courant Research Centre Geobiology, Georg-August-University Göttingen, 37077 Göttingen, Germany; ^3^Federal Institute for Geosciences and Resources, 30655 Hannover, Germany; ^4^Department of Geobiology (Geoscience Institute), Georg-August-University Göttingen, 37077 Göttingen, Germany; ^5^UK Centre for Astrobiology, School of Physics and Astronomy, University of Edinburgh, Edinburgh EH9 3JZ, UK

For a long time, Archaea have been considered as an ancient prokaryotic group comprising specialists restricted to narrow ecological niches. This opinion might have been supported by the characteristics of the first well-investigated isolates, being strictly anaerobic (methanogens), halophilic (haloarchaea), or thermophilic (various groups). This is, however, just the tip of the iceberg. Archaea are abundant in all ecosystems. Representatives of the whole domain span the widest range of ecological adaptations from psychrophilic to hyperthermophilic. They tolerate the widest range of pH as well as salt concentrations and use all types of substrates comprising all kinds of organic molecules as well as reduced inorganic compounds. 

Nowadays, a multitude of experimental tools is available allowing researchers to study the important role of Archaea and other microorganisms in environmental processes. Most important, analysis of environmental DNA and RNA opens a direct way to assess diversity in specific habitats. The pioneering findings of Carl R. Woese for assessing microbial taxonomy by ribosomal RNA sequence analysis and comparison, together with recently developed powerful amplification and sequencing techniques, widely opened the doors for detection of new hitherto unknown archaeal groups. In particular, the phyla Crenarchaeota and Thaumarchaeota and their significance in environmental cycles could be determined by these techniques. Other tools gain in importance for the assessment of environmental processes. With the increasing availability of annotated genomes, the analysis of metagenomes and metaproteomes becames possible and now helps us to understand the metabolic processes and networks within microbial communities. The identification of lipids allows bulk quantification of specific prokaryotic groups in recent habitats and even in fossil settings. Finally, microscopic techniques along with functional analysis helps us to understand some of these environmental processes also at structural levels, for example, in microbial mats.

This special issue of the journal Archaea addresses several aspects of the metabolic, structural, and ecological diversity of Archaea from a wide range of research fields. 

The contributions by B. Wemheuer et al. point out the diversity of Bacteria and Archaea in hot springs from the Kamchatka Peninsula, revealing the dominance of Proteobacteria, Thermotogae, and Thaumarchaeota. In a second publication, B. Wemheuer et al. address the diversity in a marine habitat with haloarchaea (Halobacteria) as the predominant archaeal group. One of the key processes in sediments is methanogenesis. M. R. Torres-Alvarado et al. show in their study the changes in the abundance and activity of methanogens during dry and rainy seasons in tropical estuarine sediments. The contribution of L. A. Fernández-Güelfo et al. addresses the directed processes of methanogenesis in an anaerobic reactor suitable for wastewater treatment. The whole anaerobic food chain at different temperatures was evaluated by A. Siggins et al. in an anaerobic reactor with trichloroethylene as a substrate. X. L. H. Lim et al. used the lipid archaeol in anoxic water saturated soil as a biomarker for the biomass of methanogens. Another marker for methanogens was evaluated by C. Wrede et al. for microbial biofilms. Methyl coenzyme M reductase was detected in situ by respective antibodies and was found to be a suitable indicator for the enzymes catalyzing methanogenesis as well as the reverse pathway anaerobic oxidation methane (AOM). Another research article by C. Wrede et al. addresses iron biomineralization in the course of AOM. Finally, a review article on Archaea in symbiosis briefly summarizes the diverse symbiotic interactions of Archaea with organisms of all other domains. 

We hope that this issue will support to the statement stressed previously: Archaea, being as diverse as Bacteria, make their contribution to all important global elemental cycles.

*Michael Hoppert* *Martin Krüger* *Joachim Reitner* *Charles Cockell*

